# The effects of aromatherapy massage on improvement of anxiety among patients receiving palliative care: A systematic review of randomized controlled trials

**DOI:** 10.1097/MD.0000000000014720

**Published:** 2019-03-01

**Authors:** Chia-Hsien Hsu, Ching-Chi Chi, Pei-Shih Chen, Shu-Hui Wang, Tao-Hsin Tung, Shih-Chung Wu

**Affiliations:** a Department of Public Health, College of Health Science, Kaohsiung Medical University, Kaohsiung; b Department of Dermatology, Chang Gung Memorial Hospital, Linkou, Taoyuan; c College of Medicine, Chang Gung University, Taoyuan; d Department of Dermatology, Far Eastern Memorial Hospital; e Graduate Institute of Applied Science and Engineering, College of Science and Engineering, Fu Jen Catholic University, New Taipei; f Department of Medical Research and Education, Cheng Hsin General Hospital, Taipei; g Department of Surgery, Kaohsiung Chang Gung Memorial Hospital, Kaohsiung, Taiwan.

**Keywords:** anxiety, anxiety disorder, aromatherapy, emotional disorder, essential oil, hospice care, palliative care, psychological disorder

## Abstract

**Background:**

Anxiety in patients receiving palliative care is a noteworthy concern because it may affect their quality of life. Aromatherapy has been widely utilized to improve anxiety among patients receiving palliative care.

**Objective:**

To investigate the effectiveness of anxiety improvement in patients receiving palliative care by comparing the intervention group (aromatherapy massage) with the control group (common massage alone).

**Methods:**

A literature search was performed using PubMed, Cochrane Library, Embase, MEDLINE, and CINAHL for all related studies from inception through November 30, 2018 without restriction on language. A quantitative synthesis of randomized controlled trials (RCTs) was conducted to compare the difference in effectiveness scores between the aromatherapy massage and only common massage groups by employing a random-effect model.

**Results:**

We included three RCTs with a total of 160 participants (81 in the intervention group and 79 in the control group) in our systematic review and conducted a quantitative synthesis. The secondary data from the reviewed trials were then pooled using a random-effect model. Anxiety (mean difference = −2.60 [95% confidence interval: −7.82, 2.63], *P* = .33) was assessed using anxiety scores from the State-Trait Anxiety Inventory.

**Conclusion:**

Compared with common massage alone, aromatherapy massage does not provide significant effectiveness of anxiety improvement among patients receiving palliative care.

## 1 Introduction

Psychiatric and psychosocial disorders among patients with cancer have been viewed as a major consequence of the disease and its treatment.^[1]^ For the clinical viewpoint, there are 6 clinical types of psychological reactions, that is, dependency, anxiety, postoperative depression, hypochondriac response, obsessive-compulsive reactions, and paranoid reactions. Anxiety is one of them commonly observed after cancer diagnosis and further treatment procedure.^[2]^ The prevalence of anxiety among patients receiving palliative care vary greatly according to studies on patient populations, the applied diagnostic criteria, and the assessment method (i.e., self-reports vs structured interviews).^[3]^ A recent meta-analysis reported that the pooled prevalence of anxiety disorders was estimated 9.8% (95% confidence interval [CI]: 6.8–13.2%) according to 6 studies conducted under palliative care settings in the United States and United Kingdom.^[4]^ Anxiety in patients receiving palliative care is a noteworthy concern because it may adversely affect their quality of life.^[5,6]^

Complementary and alternative medicine (CAM) is progressively evolving in developing and developed countries worldwide. Aromatherapy is one of the fastest growing CAM therapies and is widely utilized for patients with cancer to alleviate their discomfort within cancer care settings.^[7]^ Aromatherapy is practiced in 2 forms: massage with essential oil and direct inhalation of essential oil. Aromatherapy massage has been reported to relieve self-reported anxiety symptoms immediately after the therapy, and patients consider aromatherapy massage to be positive and beneficial.^[8–11]^ Aromatherapy administered through inhalation of oils without a massage does not appear to reduce anxiety.^[12]^

Aromatherapy massage is related to clinical benefits up to 2 weeks after intervention.^[13]^ However, it is often compared with control groups (typical supportive care alone) instead of common massage alone. We were unable to determine whether the effectiveness of anxiety improvement in patients receiving palliative care was caused by essential oil or massage. Therefore, we conducted a systematic review of randomized controlled trials (RCTs) to compare the effects of the intervention of massage with essential oil with that of common massage alone.

## 2 Materials and methods

### 2.1 Data source and searches

We conducted a systematic search of RCTs that compared the interventional effectiveness of massage with essential oil with that of common massage alone on anxiety among patients receiving palliative care. The literature search was conducted using PubMed, Cochrane Library, Embase, MEDLINE, and Cumulative Index to Nursing and Allied Health Literature (CINAHL) for all related trials published from inception through November 30, 2018 without restriction on language. In addition, gray literatures were captured through other sources, such as OpenGrey and Open Access Theses and Dissertations (OATD), to avoid selection bias. A search strategy was developed for the aforementioned electronic databases, using key words in Medical Subject Headings (MeSH), namely (“aromatherapy” [Mesh] OR aromatherap∗ [Title/Abstract] OR essential oil [Title/Abstract]) AND (“anxiety” [Mesh] OR anxi∗ [Title/Abstract] OR emotion∗ [Title/Abstract] OR psycholog∗ [Title/Abstract] OR disorder∗ [Title/Abstract]) AND (“palliative” [Mesh] OR “hospice” [Mesh] OR palliat∗ [Title/Abstract] OR hospice∗ [Title/Abstract] OR care [Title/Abstract]) (Table 1). The reference lists of the screened trials or other related reviews were manually examined to further identify additional similar studies. The protocol of this systematic review was registered in the PROSPERO under the number CRD42018118105.

**Table 1:**
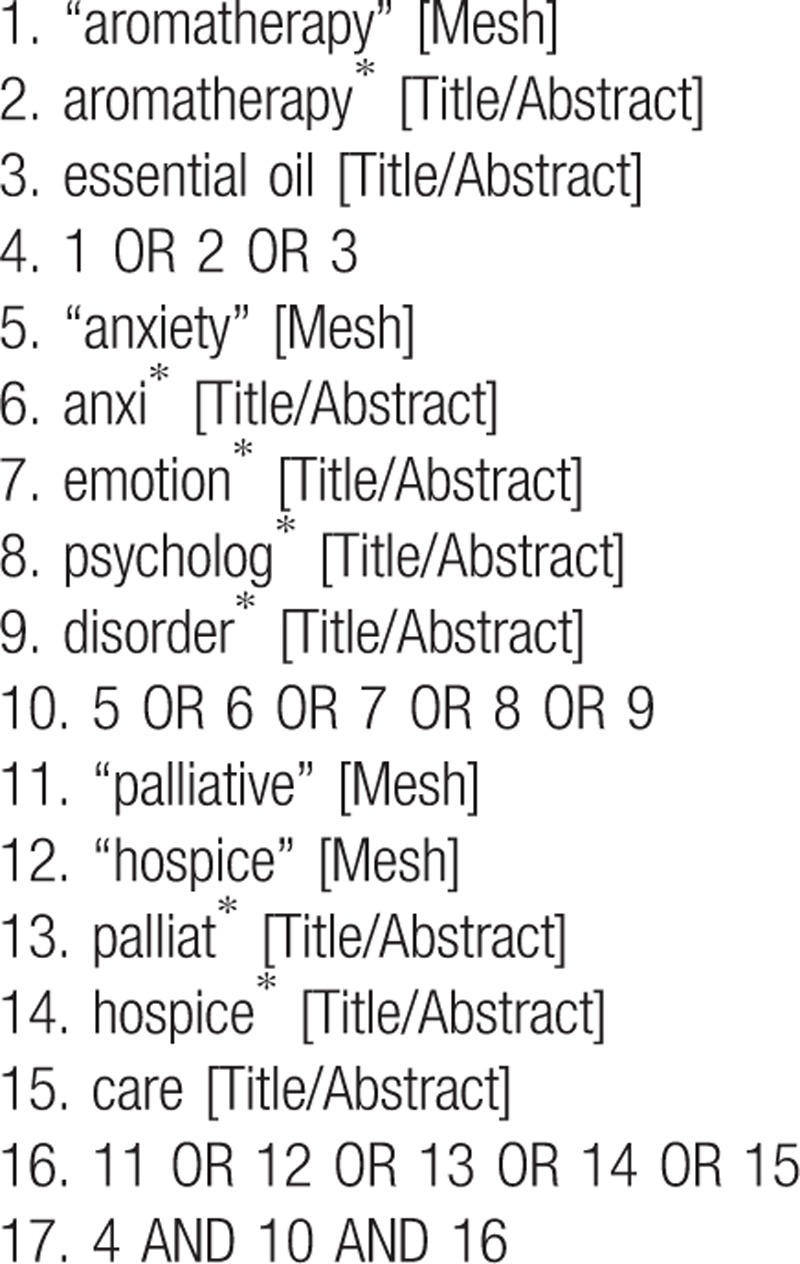
Search strategy in PubMed up till November 30, 2018 (similar search run in other databases).

### 2.2 Study selection

Two reviewers independently screened eligible RCTs that directly compared aromatherapy massage with common massage. Inclusion criteria for the studies were as follows: only anxiety as an outcome instead of including other multiple emotional disorders on a scale; complete scale mean scores plus standard deviation for pretests and posttests from 2 assigned groups, respectively. Exclusion criteria were as follows: patients receiving day-care or usual supportive care alone considered as a control group; study measures using other scales for anxiety. The full texts were checked carefully to see if there was any potentially relevant information.

### 2.3 Data extraction and quality assessment of methodology

Two reviewers independently abstracted the following characteristics of included trials based on a normalized data collection form: 1st author, publication year, study design, participant source, study population per group, intervention (aromatherapy massage and common massage), components of essential oil, follow-up duration, outcome measurement. The same reviewers independently evaluated the methodological quality of the included trials by using the Cochrane Collaboration tool. We evaluated the following 7 domains that are related to biased estimates of intervention effects: random sequence generation, allocation concealment, participant and personnel blinding, outcome assessment blinding, incomplete outcome data, selective reporting, and other biases.

### 2.4 Statistical analysis

Review Manager 5.3 (The Nordic Cochrane Centre, The Cochrane Collaboration, 2014) was used for quantitative synthesis. The outcomes of intervention effect in these 3 RCTs were defined using anxiety scores at baseline and after all interventions. Moreover, the intervention effect was summarized by using the mean difference (MD) with a 95% CI. If the information about means, standard deviation, or number of participants was unclearly reported in the articles, we would attempt to contact with the corresponding author and further understand relevant details.

As for, statistical heterogeneity, the *χ*^2^ and *I*^2^ were used for inconsistency statistics. A *P*-value <.10 was considered significant heterogeneous. Heterogeneity was stratified as absent (*I*^2^: 0–25%), low (*I*^2^: 25.1–50%), moderate (*I*^2^: 50.1–75%), or high (*I*^2^: 75.1–100%).^[14]^ A random-effect model was used because low statistical heterogeneity existed across these trials (*P* = .31; *I*^2^ = 13%).

## 3 Results

**Figure 1:**
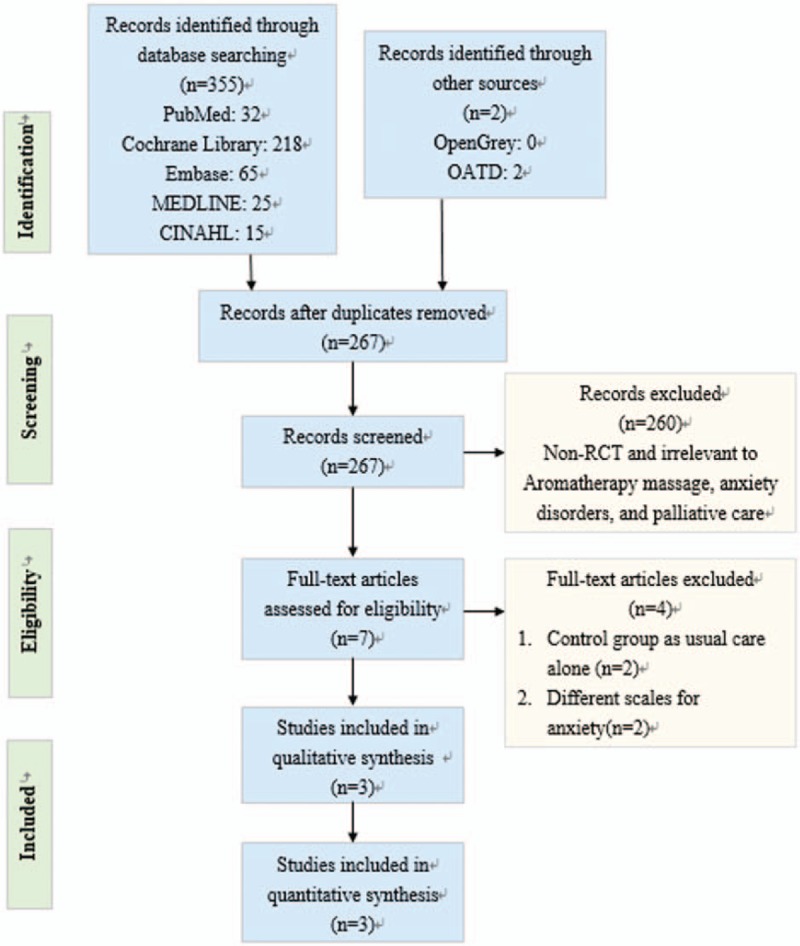
Preferred Reporting Items for Systematic Reviews and Meta-Analyses (STAI) flow diagram.

Figure 1 presents the search process and the final selection of relevant trials according to the preferred reporting items for systematic reviews and meta-analyses (PRISMA) guidelines.^[15]^ All records (n = 355) were identified through database searching. After screening the titles and abstracts and excluding 258 articles, 7 full-text articles remained. We excluded 4 ineligible studies on the basis of the exclusion criteria. Finally, 3 RCTs with a total of 160 participants were included in further analysis of quantitative synthesis.

The selected trials’ characteristics are summarized in Table 2. These 3 trials were published in 1995, 1999, and 2006, respectively. The trials included a total of 160 participants (81 in the experimental group and 79 in the control group) receiving palliative care in patients’ homes, outpatient aromatherapy clinics, and palliative care centers. The intervention group received massage with essential oil (santalum album oil and Roman chamomile oil combined with sweet almond carrier oil in the 2 trials, respectively), whereas the control group received massage with carrier oil alone. The treatment duration was at least 3 weeks, and a follow-up was conducted 1 to 4 weeks after massage course completion. All trials assessed the improvement effects on anxiety according to the State-Trait Anxiety Inventory (STAI).^[16]^

**Table 2:**
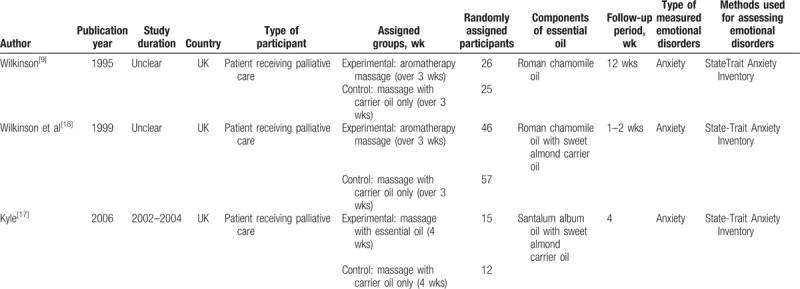
Characteristics of 3 randomized controlled trials included in this systematic review and meta-analysis.

**Figure 2:**
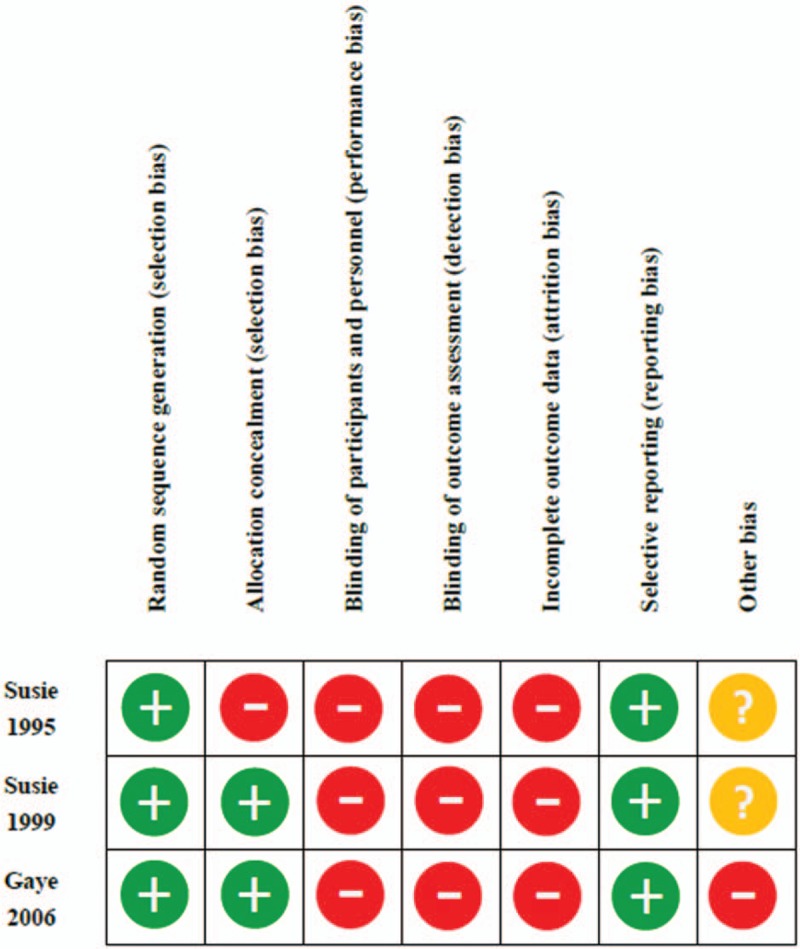
Risk of bias summary: authors’ judgments about each risk of bias item for each included study. (“+” indicates a low risk of bias; “-” indicates a high risk of bias; “?” indicates an unclear risk of bias).

For risk of bias appraisal (Fig. 2), all trials could not incorporate “blind” or “double blind” features into the study designs. A high risk of attrition bias was also stated in the discussion sections. Other potential biases were defined as the difference in medical diagnosis loading with intervention settings (e.g., in-house, clinic, and day-care), the amount of anxiolytics prescribed, and the self-selection of participants (i.e., patients who requested aromatherapy specifically to be a part of their palliative care).^[17]^

**Figure 3:**
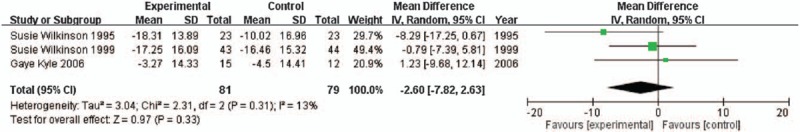
Forest plot of anxiety (State-Trait Anxiety Inventory).

We pooled the secondary data from the reviewed trials by using a random-effect model (Fig. 3). Anxiety (MD = −2.60 [95% CI: −7.82, 2.63], *P* = .33) was assessed on the basis of the anxiety scores from the STAI.^[16]^ Statistical heterogeneity was observed across the trials (*χ*^2^ = 2.31, *P* = .31, *I*^2^ = 13%).

**Figure 4:**
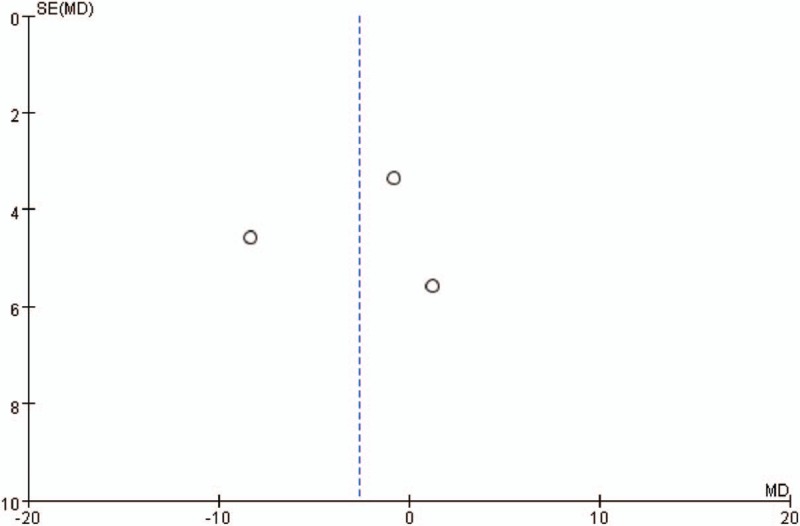
Funnel plot of anxiety (State-Trait Anxiety Inventory).

Publication bias was defined as the publication or nonpublication of studies depending on the direction and statistical significance of the results, and the 1st systematic investigations of publication bias focused on this aspect of the problem. As Figure 4, the funnel plot was symmetry, indicating no series publication bias in this study.

## 4 Discussion

### 4.1 Clinical implications

Aromatherapy massage does not appear to provide many long-term benefits for anxiety in patients with cancer compared with typical supportive care alone.^[13]^ We further investigated whether massage or essential oil improved the treatment effectiveness. We screened 3 RCTs that compared the intervention group (aromatherapy massage) with the control group (massage alone) and pooled the outcomes of anxiety. Because the sample size of each trial was not enough, we used a quantitative synthesis to combine the samples from each trial. A bigger sample size enabled the collection of conclusive and reliable results.

Wilkinson et al demonstrated significant improvement only within the intervention group (aromatherapy massage), as compared with the control group (massage alone).^[18]^ However, our summarized result showed no significant MD between the intervention (aromatherapy massage) and the control (massage alone) groups. Thus, we inferred that the improvement in the intervention group (aromatherapy massage) was mainly caused by the massage.

Because the reviewed trials did not provide complete details of the massage courses, we were unable to confirm the effect of the intensity of the techniques or strength of massage on improving effectiveness. In addition, the components of essential oil used in the intervention groups of the included trials varied. Future subgroup analyses must be conducted by collecting more relevant trials to assess whether the various fragrances are a confounding factor and to verify that the improvement is caused by common massage alone, and not by the difference in fragrance.

A limitation of the included studies was that they were double-blind for the 2 compared groups, which might have resulted in bias. Although the therapists and outcome assessors were requested to follow the guidelines established by research to conduct these trials, the substantial differences between these groups could not be avoided. Nevertheless, fulfilling a double-blind trial is difficult to achieve in practice. Another limitation involved the small number of available RCTs for this systematic review. More relevant trials that compare aromatherapy massage with common massage alone must be included to verify the improvement effect on anxiety among patients receiving palliative care.

## 5 Conclusion

Aromatherapy massage offers no significant effectiveness of anxiety improvement among patients receiving palliative care. This result should be interpreted with caution because of the limited number of trials available for review. Additional RCTs are warranted to adequately assess the effect of aromatherapy massage on patients receiving palliative care.

## Acknowledgment

The authors thank the Sunflower Statistical Consulting Company, Kaohsiung, Taiwan for statistical advice.

## Author contributions

C-HH, T-HT, and P-SC conducted the study and drafted the manuscript. S-HW and C-CC participated in the study design and performed the statistical analysis. C-CC, T-HT, and S-CW conceived the study and participated in its design and coordination. All authors read and approved the final manuscript.

**Conceptualization:** Ching-Chi Chi, Shu-Hui Wang.

**Data curation:** Shu-Hui Wang.

**Formal analysis:** Chia-Hsien Hsu, Shu-Hui Wang.

**Investigation:** Chia-Hsien Hsu.

**Methodology:** Chia-Hsien Hsu, Pei-Shih Chen.

**Project administration:** Pei-Shih Chen.

**Resources:** Pei-Shih Chen.

**Software:** Pei-Shih Chen.

**Supervision:** Shih-Chung Wu.

**Validation:** Ching-Chi Chi, Tao-Hsin Tung.

**Writing – original draft:** Ching-Chi Chi, Shu-Hui Wang.

**Writing – review & editing:** Ching-Chi Chi, Tao-Hsin Tung.
